# A deep learning based approach for classifying the maturity of cashew apples

**DOI:** 10.1371/journal.pone.0326103

**Published:** 2025-06-25

**Authors:** Moritz Winklmair, Robert Sekulic, Jonas Kraus, Pascal Penava, Ricardo Buettner

**Affiliations:** 1 Chair of Hybrid Intelligence, Helmut-Schmidt-University/University of the Federal Armed Forces Hamburg, Hamburg, Germany; 2 Chair of Information Systems and Data Science, University of Bayreuth, Bayreuth, Germany; Yantai Institute of Technology, CHINA

## Abstract

Over 95% of cashew apples are left to waste and rot on the ground. However, both cashew nuts and the often overlooked cashew apples possess significant nutritional and economic value. The cashew apple constitutes the major part (90%) of the cashew fruit, with the nut forming a modest portion (10%). Cashew nuts can be harvested and processed even after lying on the ground, but cashew apples are more delicate. Assessing the maturity status of these apples still requires human visual observation due to the challenges posed by their moisture content. Timely harvesting is crucial, as the pseudofruit is prone to microbial infections upon hitting the ground, making the process time- and labor-intensive. In this study, a Deep Learning based image classification model is presented, which can be used to automatically identify mature cashew apples. The model achieved an accuracy of 95.58% in classifying the cashew apples (immature vs. mature). Overall, the results highlight the potential of Deep Learning models for the classification of cashew apples and other fruits for precision agriculture purposes. This approach could enhance the harvesting process by enabling the utilization of the entire fruit and reducing the need for manual labor, thereby unlocking the full economic potential of the cashew tree.

## Introduction

Cashew (*Anacardium occidentale L*.) has emerged as the second most popular nut in the global market [1]. The cashew industry stands out as one of the most dynamic and lucrative cash crop sectors worldwide [2,3].

While cashew nut harvesting practices are widely spread globally [4], there is little to no information about adequately harvesting and utilizing the cashew apple [5,6].

**Fig 1:**
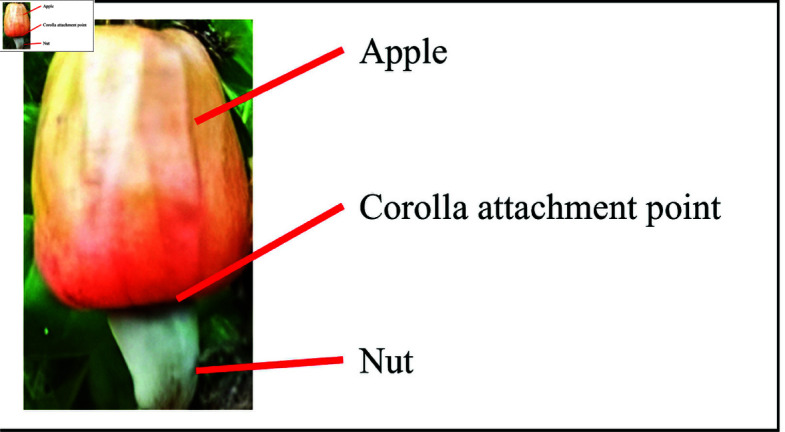
Cashew fruit.

The cashew nut (10%) makes up only a small part of the cashew fruit, while the cashew apple is the main part (90%), as shown in Fig 1 [7]. Despite this, the cashew apple, a pseudo-fruit, remains largely underutilized as a food product [8]. The apple has excellent nutritional value, six times higher in vitamin C than citrus juice [9]. Moreover, due to its high flavonoid content, it is associated with weight loss and is beneficial for diabetic patients [7,10].

In addition to the overall valuable characteristics of the cashew apple, various studies highlight the financial potential of farming, processing, and selling cashew apple products [11–13]. A wide range of value-added products can be made from cashew apples, such as juice, jam, wine, candy, and flour [14]. Additionally, the apples can also be used as animal feed and as a dietary fiber substitute in food products [15]. Because of this potential, this underutilized natural resource could undoubtedly bring additional socio-economic value to farmers and reduce poverty overall [12,16]. Developing countries’ economic success is often closely linked to the success of their agricultural sector [17], making this a substantial opportunity [5].

However, over 95% of the cashew apples are wasted and not being used for monetary valorization [18]. The major challenge for utilizing the apple is the missing information about the marketability and especially the complexity of the harvesting process [7,18–20]. When the cashew fruit (nut + apple) is mature, it falls from the tree on the ground. The nut can be harvested later [21], however, the apple needs to be discarded [21–23]. Because of high moisture, cashew apples are highly prone to rapid microbial spoilage [23]. They are metabolically active and are considered too delicate and perishable for international trade [24]. Therefore, it is of central necessity to harvest and process the apple at the optimal time [21,25].

The rise of precision agriculture leverages sensing technologies to address spatial and temporal crop variations [26,27]. Key components include UAVs, sensors, GPS, and remote sensing devices that capture real-time data on soil conditions, weather, and crop maturity [28–31]. This data-driven approach helps farmers make informed decisions and optimize harvesting practices [32]. Agronomists often visit orchards to evaluate the amount of fruits, their maturity, and their expected harvest time through visual observation, which is time and cost-intensive [33], complicating optimal maturity harvesting [21]. Therefore, integrating automated systems for accurate and efficient fruit maturity classification is essential [34,35]. Machine- and Deep Learning offer promising solutions for this automation. Machine Learning technologies have significantly impacted precision agriculture [36], employing techniques like Random Forest, Support Vector Machine, and K-Nearest Neighbor for various applications [31,37–39]. Studies show significant accuracy improvements with Machine Learning models, such as 66.5% for palm fruits, 90.35% for tomatoes, 97.69% for mangoes, and 97.99% for loquats [40–43]. While traditional statistical methods achieve satisfactory accuracy, Deep Learning, particularly Convolutional Neural Networks (CNNs), promise even higher accuracy. CNNs have broad applications in agriculture and reduce human labor [44–47]. Several applications have reached impressive accuracies: 90% for dragon fruit, 91.25% for bananas, and up to 100% for papayas and hawthorns [48–51]. These advancements modernized traditional farming practices and enhanced the accuracy and efficiency of fruit maturity classification, often surpassing manual methods, with CNN-based approaches demonstrating exceptional accuracies.

**Fig 2:**
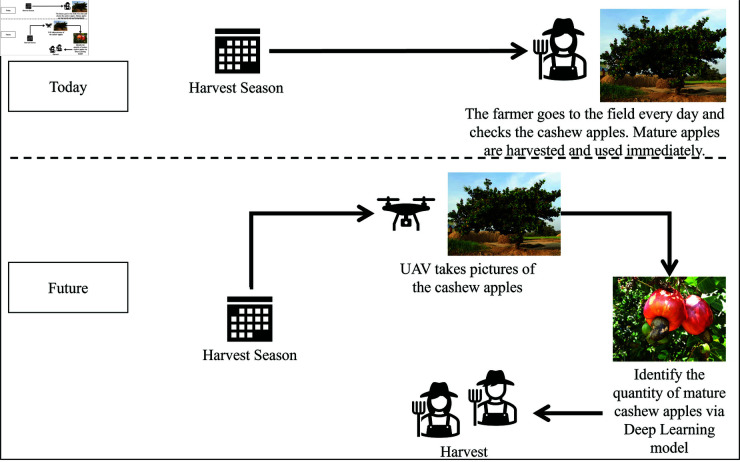
Use Case: Assisting cashew apple farmers using an UAV.

Despite these advances, images taken for precision agriculture often contain noise due to factors such as the atmospheric environment and utilized equipment like UAVs, giving the need for model optimization [52–54]. However, there is a notable gap in research regarding the maturity classification of cashew apples using innovative Deep Learning approaches. While recognizing the valuable contributions of related work, there remains untapped potential in applying similar methodologies to cashew apples in order to improve accuracy benchmarks within this specific use case. Therefore, this study aimed to develop a novel Deep Learning based model for classifying cashew apples as mature or immature. By leveraging a adapted innovative Deep Learning architecture, the goal was to enhance model performance and contribute to optimizing cashew apple yields (see Fig 2).

The most important contributions are as follows:

1. A robust Deep Learning based model was developed that is capable of accurately classifying the maturity of cashew apples with an average accuracy of 95.58%.
2. The model is a first step towards maximizing the value of cashew trees by enabling farmers to automatize cashew apple harvesting.

The rest of the paper is structured as follows. In the next section, the application of Machine Learning technologies in precision agriculture is described and existing approaches for classifying the maturity of fruits are analyzed. The details of the created model, hyperparameters, fine-tuning process, evaluation method, preprocessing and augmentation techniques, as well as the dataset used are presented in the Methodology section. An analysis of the results of the model, ensues in the following section. The discussion follows afterwards, interpreting the results. The paper is concluded in the last section, summarizing the key aspects of this study, presenting the limitations of the work, and underlining opportunities for future research.

## Research background

### Machine learning in precision agriculture

Precision agriculture, also recognized as site-specific crop management, relies on adeptly utilizing sensing technologies to discern and respond to spatial and temporal variations in crops [26,27]. The pivotal sensing component within precision agriculture integrates technologies such as UAV, sensors, GPS, and remote sensing devices to capture real-time data on variables encompassing soil conditions, weather patterns, and crop maturity [28–31]. This comprehensive approach empowers farmers to make informed decisions, optimizing agricultural practices like irrigation and pest control to enhance efficiency and sustainability [32]. The analysis of agricultural data has been facilitated through diverse technological methodologies [55,56].

Notably, the ascendancy of Machine Learning technologies has emerged as pivotal in the domain of precision agriculture [36]. For instance, techniques such as Random Forest, Support Vector Machine, and K-Nearest Neighbor, along with statistical methods, have been utilized for a variety of applications in precision agriculture [31,37–39]. Deep Learning, as a subcategory of Machine Learning, besides the previously mentioned algorithms, has also proven to be particularly beneficial [44]. Coulibaly *et al*. [45] conducted a literature review, confirming the broad application of Deep Learning in the digitalization of agriculture. This was further supported by numerous empirical examples demonstrating the effectiveness of Deep Learning based models in reducing errors typically linked to human labor [46,47]. Intelligent agriculture management systems, plant disease and pest management detection, targeted crop spraying, and irrigation systems are only a few practical applications where Deep Learning and other Machine Learning techniques were applied [57–61]. The following section will focus on literature related to this study, which used Machine Learning to classify fruit maturity.

### Machine learning based fruit maturity classification

The challenge of maintaining the quality of fruit products throughout the supply chain necessitates rigorous quality checks aimed at preserving their inherent freshness, flavor, and safety standards [62,63]. Of paramount importance in this quality assurance process is the consideration of fruit maturity, a defining factor that significantly influences the overall quality of the product [34]. Traditionally, the evaluation of fruit maturity has been relegated to manual methods, with farmers relying on visual observation to gauge the optimal harvesting time [34]. Unfortunately, this traditional approach is not without its drawbacks, as it proves to be both time-consuming and inherently prone to errors [33,34]. Consequently, there is a need for the integration of automated systems to facilitate accurate and efficient fruit maturity classification [34,35]. Machine Learning is one potential solution to automate certain steps in the process of fruit maturity classification.

An extensive literature review, to identify the most pertinent studies in the field of fruit maturity classification using machine learning models, was conducted. The focus was identifying key methodologies, performance metrics, and innovative approaches within this domain. This focus allowed the authors to understand the broader trends in fruit maturity classification. The comprehensive review provides a solid foundation for the study. Table 1 provides an overview of the latest literature in this field. It is structured as follows: first, statistical Machine Learning methods and then methods using CNNs, are presented, predicting fruit maturity.

**Table 1:** Overview on related work.

Reference	Year	Fruit	No. of Classes	Classifier	Accuracy	UAV
Marin *et al*. [40]	2020	palm fruits	binary	NB	66.50%	yes
Goyal *et al*. [41]	2024	tomatoes	3-classes	SVM, DT, RF, GBM	90.35%	no
Prabhu *et al*. [42]	2024	mango	3-classes	RF, SVM	97.69%	no
Feng *et al*. [43]	2023	loquat	3-classes	CARS-MLR	97.99%	no
Khatun *et al*. [48]	2024	dragonfruit	binary	CNN	90.00%	no
Shuprajhaa *et al*. [49]	2023	banana	4-classes	CNN	91.25%	no
Olisah *et al*. [64]	2024	blackberry	binary	CNN	95.10%	no
Saranya *et al*. [65]	2021	banana	4-classes	CNN	96.14%	no
Chmaj *et al*. [66]	2021	oranges	3-classes	CNN	96.43%	yes
Mahmood *et al*. [67]	2022	jujube	3-classes	CNN	98.26%	no
Chang *et al*. [68]	2022	pineapple	5-classes	CNN	99.27%	no
Behera *et al*. [50]	2021	papaya	3-classes	CNN	100%	no
Azadnia *et al*. [51]	2023	hawthorn	3-classes	CNN	100%	no
Kangune *et al*. [69]	2019	grapes	2-classes	CNN	79.49%	no
Elwirehardja *et al*. [70]	2021	oil palm	6-classes	CNN	89.30%	no
Gururaj *et al*. [71]	2022	mango	4-classes	CNN	93.40%	no
Zhang & Cao [72]	2022	apple	4-classes	L-MTCNN	86.00%	no
Chen *et al*. [73]	2022	citrus	3-classes	CNN	95.07%	no

UAV - Unmanned Aerial Vehicle, NB - Naive Bayes, SVM - Support-Vector Machine, DT - Decision Tree, RF - Random Forest, GBM - Gradient Boosting Machine, CARS-MLR - Competitive Adaptive Reweighting Algorithm-Multi linear Regression, CNN - Convolutional Neural Network.

Marin *et al*. [40] applied computer vision techniques to determine the maturity stage of palm fruits using aerial images from an UAV. They implemented a Naive Bayes algorithm, which showed an overall accuracy of 66.5%. Goyal *et al*. [41] introduced an ensemble approach to predict tomato maturity. This technique combined Support Vector Machine, Decision Tree, Random Forest, and Gradient Boosting Machine regressors. The model achieved an accuracy of 90.35% in predicting the maturity stage. Prabhu *et al*. [42] focused on assessing mango fruit maturity using color and texture properties. Various features were analyzed using several classifiers, including Support Vector Machine, Random Forest, and K-Nearest-Neighbour. An accuracy of 97.69% was achieved with Support Vector Machine and Random Forests. Feng *et al*. [43] utilized hyperspectral imaging for loquat quality assessment and maturity classification. The multiple linear regression model, combined with a competitive adaptive reweighting algorithm, demonstrated an accuracy of 97.99%.

While the presented statistical methods deliver satisfactory accuracy in predicting fruit maturity, the advent of Deep Learning, especially CNNs, indicates the potential for higher accuracies [45] in predicting fruit maturity. This signals a significant step in modernizing traditional farming practices [44].

Khatun *et al*. [48] leveraged a ResNet-50 based architecture to ascertain dragon fruit maturity and quality, achieving 90% accuracy in maturity classification. Also, Shuprajhaa *et al*. [49] introduced a Deep Learning method for the classification of banana maturity. They proposed a CNN-XgBoost model that combined CNNs with eXtreme Gradient Boosting. Linear Discriminant Analysis was utilized to enhance performance with a smaller dataset. The approach achieved an accuracy of 91.25%, outperforming Support Vector Classifier, Gaussian Naive Bayesian Classifier, and K-Nearest Neighbours algorithms. Another Deep Learning approach was shown by Olisah *et al*. [64]. They introduced a novel approach to address the challenge of maturity estimation in blackberry fruits, which lack obvious visible maturity cues. They propose a multi-input CNN ensemble classifier trained on images acquired with a stereo sensor using visible and near-infrared spectral filters. The model, based on a pre-trained VGG16 network, achieved 95.10% accuracy on unseen data and 90.20% accuracy under in-field conditions. Saranya *et al*. [65], in a similiar approach, utilized a CNN architecture to classify the maturity of bananas. Their approach involved comparing a custom CNN model against advanced models through transfer learning, employing both original and augmented images for training. The custom CNN model achieved an accuracy of 96.14%. Also Chmaj *et al*. [66] proposed a method for automating fruit evaluation in orchards using Deep Learning algorithms. Here they analyze UAV images of trees and used CNNs to classify fruit maturity based on surface color and shape features, achieving 96.43% accuracy. Also, Mahmood *et al*. [67] investigated the use of two CNN models, AlexNet and VGG16, through a transfer learning approach for classifying jujube maturity. They achieved the best accuracy of 98.26% with VGG16, while Chang *et al*. [68] presented a solution for automating the detection of mature pineapples. They proposed a CNN based architecture achieving over 99.27% accuracy. In another work, Behera *et al*. [50] focused on classifying papaya fruit maturity using a transfer learning approach, with VGG19 achieving 100% accuracy, and also Azadnia *et al*. [51] reached 100% validation accuracy but for hawthorn fruit grading with Deep Learning, using Inception-V3. Kangune *et al*. [69] employed a combination of CNN and Support Vector Machine to estimate the maturity of grapes. Gaussian preprocessing was applied to the images to reduce noise and enhance feature detection. The CNN based model achieved a classification accuracy of 79.49% in determining grape maturity. For the estimation of maturity levels of citrus fruits in natural environments, Chen *et al*. [73] integrated a CNN with visual saliency maps. The approach involved a two-stage process: detecting the citrus fruits on the trees and classifying their maturity. The model achieved an accuracy of 95.07%.

In contrast, two research papers included image preprocessing with Gaussian filtering. Elwirehardja *et al*. [70] focused on using Deep Learning approaches to classify the ripeness of oil palm fresh fruit bunches on mobile devices and achieved an accuracy of 89.3%. The preprocessing stage included Gaussian filtering to smooth the images and reduce noise. Gururaj *et al*. [71] presented an automated mango grading system using Deep Learning to improve accuracy and efficiency over manual inspection methods. The system utilized CNNs to extract the mango maturity stage, achieving an accuracy of 93.40%. In this system, image pre-processing involved converting RGB images to grayscale and applying an 11x11 Gaussian blur filter to reduce noise.

Zhang & Cao [72] have used apples, a similar fruit to cashew apples, in their work. They developed a multi-task CNN for maturity classification and defect detection based on images of apples. The sub-network, which classifies the apples into four maturity levels, achieved an average accuracy of 86%, outperforming other standard architectures in this task.

From the above analysis across various studies, it’s evident that the application of Machine Learning and Deep Learning techniques enhances the accuracy and efficiency of fruit maturity classification, surpassing traditional manual methods.

Notably, only a limited number of studies utilized images from UAVs to determine the maturity of fruits. However, UAVs offer a practical solution because of their versatility in various agricultural scenarios [74]. Nevertheless, they also present certain disadvantages, as they can be adversely influenced by weather conditions, which could compromise image quality [74,75]. While being cost-effective for remote sensing, they often suffer from noise-related content loss, giving the need for model optimization [53].

Zooming in on the specific context of cashew apples, the imperative to harness the advantages presented by Deep Learning and UAVs becomes apparent. Integrating CNNs into the automated classification of cashew apple maturity stands as a promising avenue for enhancing agricultural practices and optimizing harvest schedules. To adjust the potential noise in images taken by UAVs Transfer Learning could be a promising approach. By embracing these technological advancements, farmers could not only mitigate the inherent limitations of manual assessment but also elevate the quality of their cashew apple yields. To the best of the author’s knowledge, there have been no studies presented on using Machine Learning techniques to assess the maturity of cashew apples. Therefore, new studies are essential to fill this existing gap and set a benchmark for their maturity classification.

## Methodology

To determine the maturity of cashew apples, a ResNet-50 based architecture was used. The model’s architecture is introduced in the first section. The following section provides an overview of the hyperparameters used followed by the fine-tuning process. The evaluation methods for assessing the algorithm’s effectiveness are detailed afterwards. Essential preprocessing and data augmentation techniques for optimization are covered in the following section. Lastly, the dataset used in this study is outlined.

### Model architecture

The ResNet-50 architecture, known for combining simplified optimization and very deep networks through residual functions, was chosen due to its ability to produce accurate results [76]. As shown in the research background, studies have revealed that the ResNet architecture is also a promising approach for fruit maturity classification [48,51]. A transfer learning approach was applied to leverage the knowledge embedded in pre-trained networks. Utilizing weights and biases from the ImageNet visual recognition dataset, the pre-trained ResNet-50 model provided a robust foundation for feature recognition [77]. This pre-trained model offers layers that are applicable to a wide range of real-world scenarios. The final classifier was adapted to the specific use case of this study. To enable comparability of the chosen architecture with other recent state-of-the-art deep learning architectures, the exact same methodology was used to test two other architectures in addition to ResNet-50. Inception-V3 [78] and EfficientNet-B0 [79], two current, frequently used architectures, were utilized. Only the ResNet-50 block was replaced by the corresponding EfficientNet-B0 and Inception-V3 block. The main evaluation metrics were then compared between the models.

**Fig 3:**
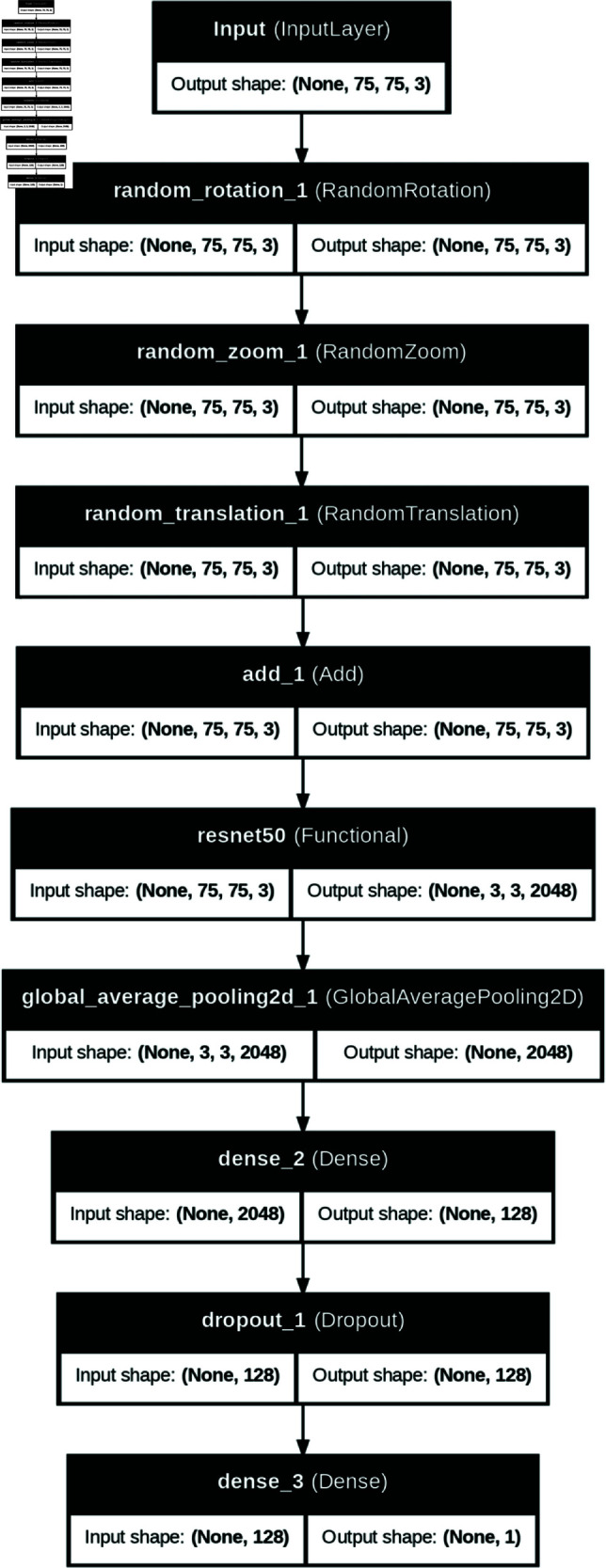
Model Architecture.

Building on this foundation, the input size of the images was configured to 75x75 pixels, accommodating 3 color channels (red, green, blue). The size of 75x75 pixels was used because the cropping of the images based on the bounding boxes resulted in very small images. Three data augmentation layers were integrated into the model ensuring that data augmentation is applied dynamically during training, further improving the model’s performance and generalization. The images were augmented by applying random zoom, random rotation and width and height translation provided by Keras library. To adapt the model for binary classification and prevent overfitting, additional layers were added to better fit the data. Initially, a Global Average Pooling 2D layer was introduced to condense the feature maps into a more compact form and increase the computational efficiency [80]. A dense layer with 128 units and rectified linear unit activation was then appended as a fully connected neural layer. Subsequently, a dropout layer was incorporated to counter overfitting and encourage the model to acquire more robust features [81]. To finalize the architecture a dense layer with a single neuron and sigmoid activation, tailored for the binary classification task was included [82]. This final layer outputs a probability [83] which serves as the basis for classifying the maturity of the cashew apples. The progression from a sophisticated pre-trained base to an adapted endpoint exemplifies a strategic blend of leveraging existing knowledge. Fig 3 gives an overview of the model used.

### Hyperparameter

The model was tested over 20 trials with various hyperparameters chosen randomly using the Keras random search tuner. The optimal hyperparameters identified from these trials were chosen for the final model for each fold in the cross validation. Table 2 details the range of hyperparameters tested and the stepsize selected for the model’s training.

**Table 2:** Optimized hyperparameters with applied value range and stepsize.

Hyperparameter	Value range	Stepsize
Random Rotation	0.05 - 0.2	0.05
Random Zoom	0.05 - 0.2	0.05
Random Translation (height)	0.05 - 0.2	0.05
Random Translation (width)	0.05 - 0.2	0.05
Dense Units	64 - 128	32
Dropout	0.0 - 0.3	0.1
Learning Rate	1e-3, 1e-4, 1e-5	Random Choice

The training process was configured to execute for a maximum of 100 epochs, and the epochs for fine-tuning were set to 30. For both initial training and fine-tuning, early stopping was applied if the validation loss did not improve for 10 consecutive epochs.

### Fine-tuning

To optimize the performance of the model and adapt it specifically to cashew maturity classification, a fine-tuning phase was carried out after the initial training with the ImageNet dataset weights. The entire model was unfrozen, allowing all layers to adapt to the classification task. The hyperparameters such as batch size, dropout rate, and dense units remained unchanged from their optimal values determined during the initial training phase. However, the learning rate was reduced to 1e-6, allowing finer tuning of the model parameters and improving convergence during training. This approach was chosen to utilize the previously learned representations while adapting the model to better fit the nuances of the cashew apple dataset.

### Evaluation method

To evaluate the performance of the model, a 5-fold cross-validation approach was used. This method involves dividing the dataset into five equal parts, using four parts to train the model and the remaining part for validation [84]. A stratified cross-validation was performed to ensure that the representation of the class distribution in each split corresponds to the original dataset. In order to address the heavy class imbalance of the dataset used, a dynamically weighted balanced loss was used, whereby a weight is calculated for each class according to its representation in the dataset, on the basis of which the loss is then calculated accordingly [85]. This process was performed iteratively, ensuring that each fold served as a validation set exactly once, while the remaining folds were used for training [84]. After each iteration, the trained model was tested on the validation dataset that the model had not seen during training. The validation dataset amounts to 20% of the whole dataset and was split before the training. To provide a comprehensive evaluation of the model’s performance, the overall performance was calculated by averaging the results from each of the five iterations. This methodological approach ensures a robust and reliable assessment of the model’s predictive performance across different folds of the dataset [84]. To thoroughly evaluate the model, the confusion matrix was used alongside the accuracy, balanced accuracy, true positive rate, positive predictive value, negative predictive value, prevalence, true negative rate, area under the curve score and F1 score.

### Preprocessing

While preparing the dataset for training, several preprocessing steps were crucial to ensure data quality and consistency. One significant aspect involved the manual sorting of cropped images. This step required the observation to verify label correspondence and ensure the presence of identifiable cashew apples. In this process, a few false annotated images were found. Images labeled as immature but showing mature cashew apples (yellow or red) were relabeled as mature. Images labeled as mature but showing immature apples (green) were relabeled as immature. Images that depicted rotten or damaged apples (brown or black), were further excluded. Moreover, some images did not depict any visible features of a cashew apple or nut. Therefore, these identified images were also excluded manually. The identified images were manually observed and relabelled by two authors with 100% reliability. After sorting the images, a total of 6,715 images were available for further processing. For the subsequent steps, the images were resized to 75x75 pixels and the pixel values scaled to the range 0-1. As mentioned in section “Evaluation Method”, the whole dataset was split into 3 parts: training (72%), validation (8%), and testing (20%). Furthermore, a merging of two classes (described in section “Dataset”) led to an under-representation of the mature cashew apple images. To correct this imbalance, this class was increased simply by copying its images in the training dataset. This was applied only to the training fold at the start of each cross-validation loop to ensure that none of these copied images were included in the validation or test datasets. The validation and test datasets were left in their original distribution to accurately reflect the model’s performance with unmodified data.

### Dataset

The dataset comprises images that showcase extensive parts of cashew trees, with annotated bounding boxes around each cashew fruit. It was published in the paper ‘Coffee and cashew nut dataset: A dataset for detection, classification, and yield estimation for machine learning applications’ by Sanya *et al*. from Makerere University in Kampala, Uganda [52]. The dataset incorporates 3,086 high-resolution images that can be used for various purposes, including Machine Learning. The images were captured by an UAV equipped with a 20/48-megapixel camera over a period of nine months in several regions of Uganda [52].

They were taken during the peak harvesting season [52], which makes them suitable for the use case presented in this paper. The images’ quality is a result of attention to meteorological conditions such as sunshine, precipitation, temperature, and cloud cover [52]. Additionally, blurry and overexposed images were excluded to ensure their accuracy and utility [52].

For classification and vision detection tasks, the images were provided with bounding boxes, which are annotated with the maturity status of the cashew fruit [52].

The information about the class and position of the bounding box is described in associated label files. The You Only Look Once format was used by the authors of the dataset to specify the coordinates of the bounding box in relative pixel values, indicating the size and position of the box [52]. The classes include 6 categories: 0 for tree (5,347 bounding boxes), 1 for flower (23,169 bounding boxes), 2 for premature (21,200 bounding boxes), 3 for immature (5,347 bounding boxes), 4 for mature (7,481 bounding boxes), and 5 for spoilt (25,820 bounding boxes) [52].

To utilize the images for the classification task, the bounding boxes needed to be cropped from the original images. The information about the class and the position of the bounding box was extracted from the associated label files when processing each image. As the coordinates were given in relative pixel values, they were converted to absolute values to determine the exact position of the bounding box.

Before cropping the images, a selective filtering process was applied to ignore the bounding boxes associated with classes 0 (tree), 1 (flower), and 5 (spoilt). Given the focus of this study on the maturity status of cashew apples, classes 0 and 5 were evaluated as irrelevant, and considering that farmers inspect only the apples during harvest season, the images of class 1 were also excluded. Consequently, classes 2 (premature) and 3 (immature) were merged into a single class labeled “immature”, while class 4 (mature) remained unchanged. Additionally, the remaining bounding boxes of the selected classes, which represented relatively large areas, such as entire trees, were excluded. This was accomplished by evaluating the size of the bounding box in proportion to the original image and omitting images where either the height or width exceeds 20% of the original image’s total dimensions.

For each qualified bounding box, the corresponding image area was cropped. All cropped images with a height or width of less than 15 pixels were excluded to ensure that only images with sufficient features were used to train the model. This process resulted in a tailored and filtered dataset of cropped images.

The dataset used in this work [52] is publicly accessible under the following link: data.mendeley.com.

## Results

The model’s performance was evaluated using a five-fold cross-validation approach to ensure a robust assessment of its generalization ability. The key performance indicators of the model across the folds are summarized in Table 3.

**Table 3:** Detailed performance indicators - ResNet-50.

Fold	Accuracy	Balanced accuracy	True positive rate	Positive predictive value	Negative predictive value	Prevalence	True negative rate	AUC score	F1 score
Fold 0	94.56%	89.13%	83.33%	34.65%	99.43%	3.13%	94.93%	92.92%	0.49
Fold 1	94.86%	82.37%	69.05%	34.12%	98.97%	3.13%	95.69%	93.63%	0.46
Fold 2	94.86%	86.10%	76.74%	35.87%	99.20%	3.21%	95.46%	92.74%	0.49
Fold 3	96.65%	73.53%	48.84%	47.73%	98.31%	3.21%	98.23%	87.45%	0.48
Fold 4	96.95%	86.06%	74.42%	51.61%	99.14%	3.21%	97.69%	94.47%	0.61
**Average**	**95.58%**	**83.44%**	**70.48%**	**40.80%**	**99.01%**	**3.18%**	**96.40%**	**92.24%**	**0.51**

**Fig 4:**
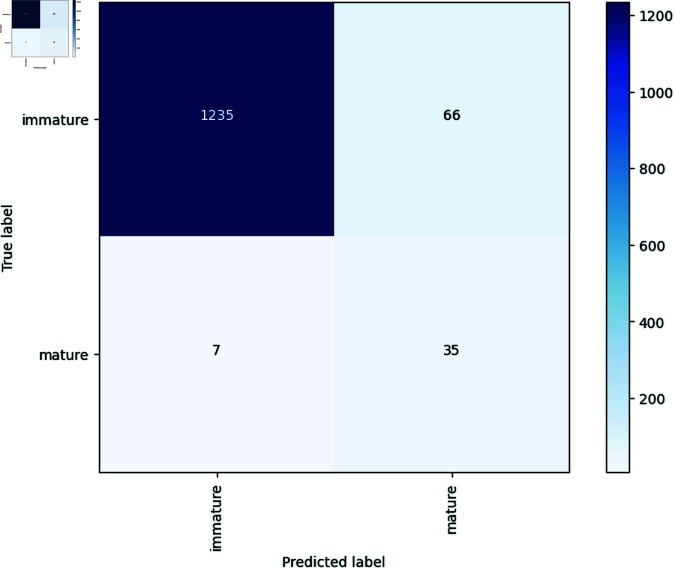
Confusion matrix.

The accuracy of the unseen test data was 95.58% across all folds. The balanced accuracy, which accounts for the imbalance in the dataset [86], averaged at 83.44%. Recall and precision are crucial metrics for evaluating the performance of a classification model, particularly in imbalanced datasets [86]. The model achieved an average recall (true positive rate) of 70.48% and a precision (positive predictive value) of 40.80% on average. The F1 score combines these two metrics and it represents a measure for imbalanced datasets that represents the ability of the model to classify positive instances while minimizing false positives and false negatives [87]. Our model achieves an F1 score of 0.51 on average over the five folds. Additionally, the model’s average negative predictive value was 99.01%, with a prevalence of 3.18%. This means that when a cashew apple is randomly selected from the dataset the probability of it being mature is at 3.18%. The specificity (true negative rate) averaged 96.40%, expressing how well the model identifies immature apples on average [88]. Lastly, the model’s Area Under the Curve (AUC) score averaged 92.24%. The AUC is a performance metric for binary classification models that represents the probability that a randomly chosen positive instance (mature cashew apple) is ranked higher than a randomly chosen negative instance (immature cashew apple) [90]. In addition, Fig 4 shows the confusion matrix of the best fold, which shows the distribution of the classifications.

## Discussion

As stated in the preceding section, the model exhibited a good ability to classify the maturity of cashew apples with a high average accuracy of 95.58%. Most of the research from the research background that have also dealt with the maturity classification of fruits achieve a similar accuracy to this paper, which is why a comparable foundation was created. However, to the best of the authors’ knowledge, no other research work classifies the maturity of cashew apples. Thus, the achieved accuracy serves as the new benchmark in this comparison. Of the fruits used in the related works, apples are visually closest to cashew apples. In their work, Zhang & Cao [72] successfully classified the maturity of apples into four classes with an accuracy of 86.00%. Even if no binary class problem is addressed in their work, the comparison of the average balanced accuracy of 83.44% achieved here with the 86.00% shows that the new approach performs comparably to other state-of-the-art approaches.

Since an imbalanced data set was used in this work, balanced accuracy is at least as important as accuracy. This metric can be used to show that the model works for both classes, mature and immature, and can make correct decisions. The average balanced accuracy of 83.44% and the confusion matrix in Fig 4 show that the model works for both classes. The same is also shown by the average F1 score of 0.51. At a maximum of 1.00, the model would classify perfectly. The value of 0.51 shows that the model is still good at achieving correct positive classifications while minimizing false positives and false negatives. Nevertheless, it is also clear that there is still room for improvement. The confusion matrix shows that the model incorrectly classifies only 66 out of 1301 immature cashew apples as mature, while it misclassified seven out of a total of 42 mature apples as immature. In percentage terms, the error of the second type therefore occurred significantly more frequently. One possible explanation is that there are simply more images of immature cashew apples in the data set and the model can learn better from them. Thus, a more extensive data set would be needed that also contains more images of mature cashew apples in order to improve the prediction there as well. The mistake of classifying an already mature apple as immature would mean that this apple could rot and is therefore no longer of any use. Conversely, an apple incorrectly classified as mature could falsify the yield estimate, for example. So both types of error are important and should be minimized for real world use. The very good average AUC score of 92.24% shows that the model does not make correct decisions by chance, but can actually distinguish well between mature and immature cashew apples [90].

Table 4 shows the comparison of the evaluation of the presented approach with different state-of-the-art deep learning architectures. The evaluation metrics that consider the class imbalance are shown. The table shows that the architecture chosen in this work (ResNet-50) achieves the best values in the comparison in almost all evaluation metrics. Compared to the Inception-V3 architecture, better results are achieved for all metrics, while EfficientNet-B0 achieves better values for the true positive rate and the negative predictive value. However, the results of the summarizing metrics such as the balanced accuracy and the F1 score, which include both classes and also all types of errors, show that the ResNet-50 architecture achieves better results overall than the EfficientNet-B0 architecture, which makes this architecture the best choice for the given problem. The potential of the applications is therefore given and should definitely be investigated further.

**Table 4:** Comparison of the approach with different state-of-the-art Deep Learning architectures.

Architecture	Balanced accuracy	True positive rate	Positive predictive value	Negative predictive value	True negative rate	AUC score	F1 score
Inception-V3	64.94%	33.69%	21.86%	97.80%	96.19%	71.46%	0.27
EfficientNet-B0	78.75%	**87.79%**	9.12%	**99.42%**	69.72%	86.49%	0.17
ResNet-50	**83.44%**	70.48%	**40.80%**	99.01%	**96.40%**	**92.24%**	**0.51**

### Practical implications

The use of UAV imagery in this study illustrates the transformative potential of aerial data collection in agriculture. UAV applications surpass traditional methods in efficiency, demand less labor, and have the capability to survey extensive areas [89]. In addition, the cost-effectiveness of UAVs [89] makes them particularly suitable for farm smallholdings. The use offers advantages in terms of scalability and detailed monitoring.

Despite the possible upside associated with integrating UAVs into agricultural practices, the application can also entail challenges. Achieving high-quality images consistently with UAVs can be demanding due to various factors, such as technical settings, environmental conditions, and crop characteristics beyond the operator’s control [91]. Consequently, establishing robust protocols for image capture is essential [91]. Another aspect to consider when employing UAVs is the incorporation of practical applications. Rather than directly embedding these into the UAVs, one alternative is to employ cloud-based solutions. Using such applications to process captured images is a viable solution that eliminates the need for lightweight network architectures in mobile applications [92].

This technological evolution in agriculture aligns with the increasing economic potential of cashew apples [11–13].

By advancing the understanding of how Deep Learning can be applied to cashew apple maturity classification, the authors are paving the way for more efficient and accurate harvesting strategies. Such advances are not just academic but have real-world implications. By more accurately identifying maturity, farmers can better plan their harvesting processes to ensure optimal quality and yields of cashew apples [93]. This, in turn, supports sustainable agricultural practices by minimizing waste and increasing productivity, in line with the broader goals of improving food security and sustainability in the cashew industry [3,7]. In a real-world application, the approach developed in this work could be used in a higher-level system in which cashew apples are first detected with an object recognition module and then reliably classified with the architecture shown on the basis of their maturity.

## Conclusion

A highly accurate Deep Learning based model for classifying the maturity of cashew apples is introduced in this study. Achieving an average accuracy of 95.58% and a balanced accuracy of 83.44%, the ResNet-50 based model employs transfer learning and incorporates custom layers to mitigate overfitting and enhance generalizability. To counteract training data imbalance, up-sampling was applied, while data augmentation methods increased training data diversity. The effectiveness of the model was validated through k-fold cross-validation, testing with an unseen dataset, and comprehensive evaluation of various performance metrics [84].

To the best of the authors’ knowledge, this is the first time such a methodology has been deployed to assess cashew apple maturity, highlighting its novelty and potential impact and setting a new benchmark for this use case. Applying such a model in real-world scenarios could be of enormous benefit to cashew apple farmers, as they would no longer have to rely on visual observation [13]. This could ultimately lead to an increase in extracting the potential of the cashew tree. The model presented serves as a crucial step towards making this achievable.

### Limitations

Despite the presented opportunities, possible limitations have to be considered. Both technical and practical, limitations are presented.

In this study, the only publicly available dataset containing images of cashew apples on trees in orchards was utilized. While this dataset was instrumental for this study, it was constrained by the low resolution of the cropped images and some mislabeled images. This led to a reduction in the dataset size and possibly influenced the overall performance of the model [94]. The low resolution may have also limited the models’ ability to discern complex patterns and features [95].

Despite the practical focus of this study, the model was not tested for real-world applicability. For deployment, it could be beneficial to test the model within a comprehensive precision agriculture system [96]. Lastly, the evaluation of the models relied solely on the single dataset available, without validation against new datasets or real-world scenarios (i.e. pictures from various sources). This absence of external validation raises concerns regarding the robustness and generalizability of the model beyond the specific dataset utilized in this study. Hence, it is recommended to explore further approaches by testing this model and developing alternative models using additional datasets.

### Future work

There are some possible opportunities to further evaluate the practicality of the presented model and to explore the use of UAVs and CNNs in cashew orchards.

To address some limitations of this study, additional datasets need to be acquired. These datasets should contain higher resolution images and be carefully labeled to ensure accuracy and avoid significant imbalances.

Furthermore, this work introduced the classification of maturity states. To create a practical application, it would be necessary to develop a detection model in combination with the classification problem. Future research could explore approaches such as You Only Look Once for this purpose [97].

In addition, the authors believe that it would be highly beneficial for farmers to develop a prediction model that predicts the optimal harvest time of immature cashew apples. This approach would refine resource management on farms, potentially resulting in cost savings and enhanced operational efficiency [98]. Therefore, future work could also focus on creating datasets with timestamps and corresponding labels, as well as developing predictive models.

In conclusion, despite its limitations, this study has made an important contribution by demonstrating that Deep Learning techniques can have valuable practical applications in the field of cashew apples. This merits further exploration to offer farmers effective solutions and to pave the way for the growing importance of the valuable cashew apple.
